# Antioxidant and Anti-Inflammatory Activities of Probiotic Strains

**DOI:** 10.3390/ijms27021079

**Published:** 2026-01-21

**Authors:** Olga Adriana Caliman-Sturdza, Josiana A. Vaz, Ancuta Veronica Lupaescu, Andrei Lobiuc, Codruta Bran, Roxana Elena Gheorghita

**Affiliations:** 1 College of Medicine and Biological Sciences, Stefan cel Mare University of Suceava, 720229 Suceava, Romania; olga.caliman-sturdza@usm.ro (O.A.C.-S.); ancuta.lupaescu@usm.ro (A.V.L.); andrei.lobiuc@usm.ro (A.L.); codruta.bran@usm.ro (C.B.); 2 Suceava County Clinical Emergency Hospital, 720224 Suceava, Romania; 3 Research Centre for Active Living and Wellbeing (LiveWell), Instituto Politécnico de Bragança, 5300-253 Bragança, Portugal

**Keywords:** probiotics, gut–brain axis, neuroinflammation, gut health, cytokines, oxidative stress

## Abstract

This review highlights the anti-inflammatory and antioxidant effects of probiotics and their complex health-related impacts. The main health areas targeted are gastrointestinal inflammation, neuroinflammation, systemic metabolic disorders, and liver conditions. Probiotics work mechanistically to regulate key inflammatory pathways by suppressing nuclear factor (NF-κb) and mitogen-activated protein kinase (MAPK) pathways and activating antioxidant defenses through nuclear erythroid 2-related factor (Nrf2). They stimulate anti-inflammatory cytokines (including interleukin 10 (IL-10) and inhibit pro-inflammatory mediators such as tumor necrosis factor-α (TNF-α), partly through the regulation of T cells. Probiotics also produce antioxidant metabolites (e.g., exopolysaccharides and short-chain fatty acids), which enhance the host’s resistance to oxidative stress. Supplementation with probiotics improves intestinal inflammation and oxidative injury in gut disorders. Clinical trials suggest that probiotic supplements may reduce neuroinflammation and oxidative stress, while improving cognitive or behavioral outcomes in neurodegenerative disorders. Overall, this review underscores that probiotics have potent anti-inflammatory and antioxidant effects within the gut–brain axis and across various organ systems, supporting their use as valuable adjunctive therapies for inflammatory and oxidative stress-related conditions. It further emphasizes that additional mechanistic research and controlled clinical trials are essential to translate these findings into the most effective therapeutic strategies.

## 1. Introduction

Probiotics are live microorganisms that provide health benefits when consumed in sufficient amounts by the host [1]. Friendly microbes have traditionally been used in fermented foods and as gut health supplements, and have met rigorous requirements (including acid/bile resistance, adherence, and safety) to be considered probiotics [2,3]. In addition to enhancing digestion, research results demonstrate that probiotics can regulate the immune system and oxidative homeostasis, thus fighting the inflammatory process and oxidative stress within the organism [4,5]. Chronic inflammation and redox imbalance, characterized by excessive production of reactive oxygen species (ROS), are now recognized as important contributors to the pathophysiology of numerous disorders, including inflammatory bowel disease (IBD), metabolic dysfunctions, cardiovascular diseases, and several neurodegenerative conditions [6]. However, neurodegenerative diseases, such as Alzheimer’s disease (AD), are highly heterogeneous and multifactorial. Current hypotheses of AD pathophysiology encompass amyloid-β and tau cascades, neuroinflammatory mechanisms, vascular and metabolic dysfunction, infectious or microbial factors, as well as strong genetic predispositions (e.g., apolipoprotein E (APOE) genotype and other susceptibility loci) [7]. These genetic and molecular factors may influence both disease susceptibility and host immune responses, underscoring that no single mechanism alone accounts for disease onset or progression [7,8]. Within this complex framework, probiotic interventions have been proposed as supportive and modulatory strategies rather than universal preventive solutions.

Probiotics have been shown to contribute to immune homeostasis and antioxidant defenses in various experimental and clinical contexts [9,10]. It is interesting to note that some strains of *Lactobacillus* (a significant family of lactic acid bacteria) have been shown to exhibit strong anti-inflammatory and antioxidant effects in both in vitro and in vivo conditions [11]. Several species of *Lactobacillus* can reinforce the gut barrier, change gut microbiota composition, and generate bioactive metabolites that reduce gut inflammation [12]. Other probiotics, such as *Lactobacillus*, *Bifidobacterium*, and spore-forming bacilli, have also shown immunoregulatory and anti-oxidative stress effects [13]. This review will give an overview of the anti-inflammatory and antioxidant properties of the probiotics, specifically *Lactobacillus* spp. This mechanistic evidence (cytokine regulation, oxidative stress indicators, intestinal microbiota, gut–brain axis, intestinal epithelial integrity) summarize the results of human clinical trials and in vitro or preclinical research. The positive effect on gut health and neuroinflammation is specifically discussed, along with systemic effects on metabolic, hepatic, and other organ systems, emphasizing their overall impact on health.

The anti-inflammatory and antioxidant potential of probiotics has intrigued the scientific community, and in vitro and in vivo tests have therefore highlighted their beneficial effects on health (Table 1). These results have reinforced interest in the mechanisms by which probiotics exert these effects, going beyond their simple action at the molecular level. Understanding how probiotic strains interact with the intestinal microbial ecosystem and influence the functioning of various organs has become a central topic of modern research. Thus, an increasing number of studies are exploring how probiotics contribute to maintaining a balanced microbiota and supporting systemic health.

## 2. Methods

This comprehensive literature research was conducted to identify relevant studies for this review. Multiple electronic databases—Web of Science, PubMed, and Scopus—were searched for articles on probiotics’ anti-inflammatory and antioxidant effects. The search strategy combined terms related to probiotics with terms related to inflammation, oxidative stress, and various health conditions. For example, search queries included combinations of keywords such as “probiotics” OR “*Lactobacillus*” (to capture probiotic-related articles) AND “anti-inflammatory” OR “antioxidant” (to capture mechanisms of interest) AND terms for specific contexts like “gut–brain axis”, “neuroinflammation”, “gut health”, “cytokines”, “oxidative stress”, “metabolic disease”, “cancer”, “gut microbiota”, or “mental diseases”. The search was limited to articles published from 2016 up to November 2025 to capture the most recent findings, although earlier foundational studies were also considered for background context when relevant. We supplemented database searches with examination of reference lists from key articles to ensure no major publications were missed. All records identified through the searches were imported into a reference manager, and duplicate entries were removed. The selection process was carried out in multiple stages. In the screening stage, two reviewers (the authors of this article) independently screened the titles and abstracts of retrieved records to exclude clearly irrelevant publications. Next, in the eligibility stage, we obtained the full texts of all remaining articles and evaluated each against the predetermined inclusion and exclusion criteria. Any disagreements or uncertainties during screening or full-text review were resolved through discussion and consensus among the authors.

Inclusion criteria: Studies were included if they met all of the following conditions:

- Published in English in a peer-reviewed journal (between 2016 and November 2025).
- Addressed probiotics (particularly *Lactobacillus* species) in the context of anti-inflammatory and/or antioxidant effects.
- Provided relevant data or findings pertaining to at least one of the key focus areas of this review (e.g., gut inflammation, intestinal barrier function, oxidative stress markers, neuroinflammatory outcomes, metabolic or hepatic inflammation).
- Employed sound methodology appropriate to the study type (for example, clinical trials with control groups, or laboratory studies with proper controls), ensuring the results were of sufficient scientific quality to inform this review’s objectives.

Exclusion criteria: Studies were excluded if they met any of the following conditions:

- Not written in English, or not a full-length article (e.g., conference abstracts, editorials, or dissertations without peer review).
- Focused on topics outside the scope of probiotic anti-inflammatory/antioxidant effects (for instance, studies solely about probiotics’ effects on food preservation or unrelated microbial processes were not included).
- Lacked relevance to this review’s objectives upon full-text examination (e.g., did not actually report inflammatory or oxidative outcomes).
- Had significant methodological limitations that could bias the results (for example, sample sizes too small to draw conclusions, or lack of any control/placebo in an interventional study), rendering the findings less reliable for our analysis.

In total, the initial database search yielded a broad set of the literature (several hundred articles). After removing duplicates and screening out clearly irrelevant titles/abstracts, we assessed the full text of the remaining articles for eligibility. Through this rigorous selection process, a final set of 209 articles was included that met all criteria and were considered appropriate for addressing the objectives of our review. These articles form the basis of the narrative synthesis in the subsequent sections.

## 3. Mechanisms of Probiotic Anti-Inflammatory and Antioxidant Action

There are hypothesized pathways of the role of probiotics (particularly *Lactobacillus* spp.) in anti-inflammatory and antioxidant activity at the gut interface. The positive bacteria are also capable of enhancing the tight junction protein (TJ proteins such as occludin, claudins, E-cadherin, ZO-1) expression in intestinal epithelial cells, thus improving the gut barrier [23]. They cause the generation of protective HSP and the expression of host antioxidant enzymes (AOEs), such as superoxide dismutase (SOD), glutathione peroxidase (GSH-Px), and catalase (CAT), which prevent oxidative damage of intestinal cells [24]. Probiotics also stimulate the secretion of mucus (e.g., mucin Muc-2, Muc-4, and trefoil factor TFF3) to stiffen the mucus layer [25,26]. They regulate the number of immune cells in the lamina propria by decreasing the number of pro-inflammatory immune cells (e.g., M1 macrophages, active dendritic cells) and increasing the number of anti-inflammatory or tolerogenic ones (e.g., M2 macrophages, regulatory T cells) [27,28]. In line with this, probiotics suppress pro-inflammatory mediators such as interleukin (IL-6), IL-1 2, TNF-α, chemokines such as interferon gamma-induced protein 10 (IP-10), nitric oxide (NO), and prostaglandin E2, and promote anti-inflammatory mediators such as IL-10, transforming growth factor-α (TGF-α), and granulocyte colony-stimulating factor (G-CSF) [29,30]. Certain strains even secrete enzymes (e.g., chondroitinase, hyaluronidase) and peptides to degrade harmful bacterial products (e.g., glycosaminoglycan-degrading enzymes) or signals of inflammation and break the vicious cycle of tissue damage and inflammation [31]. All these actions contribute to the preservation of epithelial integrity and homeostasis of the immune system (inset depicts a wound situation in which probiotics come in to prevent pathogen- and toxin-induced disease). There are a variety of complementary effects of probiotics on host pathways. One of them is immunomodulation: numerous probiotic strains are capable of modulating the balance between a pro-inflammatory and an anti-inflammatory cytokine profile. An example is that a variety of *Lactobacillus* spp. (such as *L. rhamnosus*, *L. jensenii*, *L. reuteri*, *L. casei*, *L. plantarum*) have been demonstrated to downregulate major pro-inflammatory cytokines IL-6, IL-1b, TNF-α, among others, in inflamed tissues and increase the synthesis of anti-inflammatory cytokines (IL-10) [15,17,22,29,32,33]. In a clinical setting, this may reestablish a normal level of immunophenotype. For example, in patients with IBS, an abnormally low IL-10/high IL-12 response (reminiscent of a Th1-biased proinflammatory condition) was downregulated with *Bifidobacterium longum* 35624 supplements [14]. These effects are accomplished by the interaction of the probiotic bacteria with the pattern recognition receptors of host cells. A soluble protein produced by *L. rhamnosus* was discovered to bind Toll-like receptors ((TLR)4) on intestinal cells and block the MyD88/NF-oB signaling pathway, subsequently reducing the expression of inducible nitric oxide synthase (iNOS) and cyclooxygenase-2 (COX-2) and the release of the proinflammatory mediators NO and prostaglandin E 2 [15,34]. *L. casei* and *L. paracasei* have the capacity to secrete a protease, lactocepin, selective in degrading the pro-inflammatory chemokine IP-10, therefore preventing IP-10 recruitment of immune cells to the gut mucosa, leading to a markedly reduced T cell infiltration and inflammation in probiotic-treated colitic mice [16,35]. Probiotics can also trigger tolerogenic dendritic cells and stimulate regulatory T cells, which further bias immune response to anti-inflammatory [36,37]. In general, probiotics modulate chronic inflammatory processes through multiple pathways by inhibiting the excessive activation of the immune response.

Another mechanism is the enhancement of the gut barrier and decreasing the endotoxin translocation. Probiotics enhance intestinal junctional integrity between intestinal epithelial cells, which discourages the discharge of pro-inflammatory bacterial components (such as lipopolysaccharide (LPS)) into the bloodstream [38]. Tight junction proteins (ZO-1, occludin, claudin-1, E-cadherin) lost during inflammation were restored by *L. acidophilus*, *L. plantarum*, *L. helveticus*, and other similar strains in mouse models of colitis [39]. Similarly, a multi-strain probiotic (Bifico, which contains *Bifidobacterium, Lactobacillus*, and *Enterococcus* strains) improved colonic ZO-1 and occludin expression and had an ameliorative effect on experimental colitis in mice. This was consistent with previous studies in IBD patients receiving Bifico, which demonstrated mucosal healing and decreased gut permeability [40]. Probiotics also enhance the protective lining of mucus on the epithelium. As an example, *L. rhamnosus* and *L. plantarum* strains enhanced mucin (Muc-2, Muc-4) and trefoil factor (TFF3) gene expression in LPS-challenged mice, leading to an increase in mucus and protection of the gut lining against toxins [41]. *L. rhamnosus* GG also stands out by its high colonization capacity to intestinal mucin, which can possibly enable it to colonize the mucus niche and enhance the barrier [42]. Probiotics decrease systemic exposure to LPS and microbial products of inflammation, which increases the circulating tone of inflammation by coating the gut barrier [43]. In fact, there are reduced serum endotoxin levels and C-reactive protein with the use of probiotics in patients with metabolic diseases, which is due to an increase in gut barrier integrity [44]. Probiotic bacteria also offer direct antioxidant protection and augment host antioxidant capacity. There are a great number of *Lactobacillus* and *Bifidobacterium* strains that can synthesize or trigger antioxidant molecules. They stimulate host antioxidant enzymes in the gut mucosa; an example is that oral *L. plantarum* ZS62 in colitic mice increased colonic and serum levels of SOD (especially Mn-SOD), catalase, and glutathione peroxidase, which are associated with a decrease in tissue inflammation [45]. Likewise, *L. gasseri* (designated to overexpress Mn-SOD) had a substantial alleviating impact on colitis in IL-10-deficient mice to reduce neutrophil and macrophage infiltration in the colon [16]. In colitis, the antioxidant activity of enzymes (e.g., reduced SOD in tissues of IBD patients) is frequently lost [46,47]. Probiotics are useful in restoring such defenses and countering ROS, which power inflammation [48]. Other strains produce an HSP in the host cells that protects them against oxidative and stress-related damage. It is worth noting that *L. reuteri* ATCC PTA 4659 recovered the expression of HSP25 and HSP70 in the colonic epithelium of mice with ulcerative colitis, thus maintaining the cytoskeleton structure and avoiding oxidative damage of the intestinal cells [15]. The culture supernatant of *L. rhamnosus* GG was found to induce HSP25/72 of the intestinal cells through Jun N-terminal kinase (LNK) and p38 MAPK pathways, which play a role in the cytoprotective effect [49]. Along with enzyme induction, probiotics may also be able to directly scavenge free radicals and metal ions. Thus, probiotics have been reported to bind transition metals (lowering the formation of hydroxyl radicals) and to release metabolites (e.g., exopolysaccharides, peptides) with intrinsic antioxidant properties [50]. One such example is in the case of *L. reuteri* and the synthesis of ergothioneine—a potent dietary antioxidant—which, under oral administration, had antidepressant and neuroprotective effects on a mouse stress model, demonstrating that a microbially-derived antioxidant can have antidepressant and neuroprotective action in remote organs such as the brain, a behavior and effect again mediated by its metabolite, ergothioneine [51,52]. Probiotics lower the oxidative load that promotes chronic inflammation by more than one pathway.

**Figure 1:**
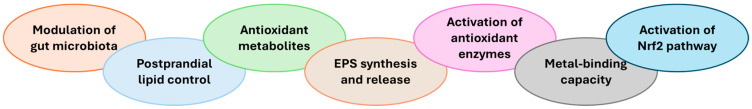
Antioxidant mechanisms of probiotics.

Numerous studies have demonstrated the antioxidant activity of *Lactobacillus* strains, attributable to both specific metabolites produced and secreted compounds (Figure 1).

For example, H_2_O_2_ produced by *L. acidophilus* promotes the oxidation of parasite proteins and the inhibition of cysteine proteases, contributing to direct antioxidant effects [53]. At the same time, secondary metabolites (organic acids, amino acids, phenols, terpenoids) obtained during fermentation activate Nrf2/ARE pathways, inhibit JAK2/STAT3/MAPK, reduce ROS levels, and modulate AT1R/PPAR-γ receptors, generating antioxidant and cytoprotective effects [54,55]. In addition, secreted or soluble metabolites (cell-free extracts) induce caspase 3/8/9 activation and regulate the Bax/Bcl-2 ratio, promoting apoptosis while scavenging DPPH radicals, showing antioxidant and antiproliferative activity on HT-29 cells [56]. Overall, these data highlight the antioxidant potential of *Lactobacillus* strains under different experimental conditions. Details regarding the antioxidant activity and antitumor properties of *Lactobacillus* and *Bifidobacterium* strains are summarized in Table 2.

Another mechanism is the regulation of gut microbial composition and metabolites. Probiotics can reestablish dysbiosis by outcompeting or suppressing pathogenic organisms and preserving the other beneficial commensal organisms. *L. plantarum* Q7 supplementation reduced the pro-inflammatory bacterial abundance (e.g., Proteobacteria) and increased the abundance of anti-inflammatory taxa in IBD models (e.g., *Bifidobacterium*, butyrate-producing families, *Lachnospiraceae*, *Ruminococcaceae*) [73,74]. The increased number of butyrate producers, in its turn, results in an increase in short-chain fatty acids (SCFA) such as butyrate, which are known to have anti-inflammatory actions in the gut (promoting T reg cells, improving epithelial nutrition). Precisely, probiotic *L. paracasei* L9 increased the number of butyrate-producing bacteria in colitic mice, which suggests collaborative activities that enhance anti-inflammatory metabolites [75,76]. Other probiotics synthesize SCFAs or other anti-inflammatory molecules (e.g., conjugated linoleic acids) or tryptophan metabolites in situ that regulate immune responses both locally and systemically [77]. All in all, probiotics enrich the beneficial microbes and metabolites and thus form a less inflammatory gut milieu.

Lastly, probiotics impact the gut–brain axis, where immune and oxidative modulations in the gut have the potential to alter the central nervous system (CNS). Probiotics may have a neural effect through vagal nerve stimulation, neurotransmitter changes, and peripheral inflammation to neuroglia [78]. For example, *L. rhamnosus* JB-1 proved to influence the γ-aminobutyric acid sub-type A (GABA) receptors’ expression in certain brain areas of the mice and reduce their anxiety/depressant behavior, which was also dependent on an intact vagus nerve as well as on CD4+ regulatory T cells [79,80]. The enzyme xanthine oxidase in the brain was also observed to be blocked by other strains (e.g., *L. paracasei* CCFM1229, *L. rhamnosus* CCFM1228), thus reducing the production of ROS associated with depressive phenotypes [81]. In an infection-related neuroinflammatory model, the *L. gasseri* NK109 administration caused a remarkable decrease in the hippocampal IL-1b and an increase in the brain neurotrophic factor (BDNF), which suggests that the probiotic reduced neuroinflammatory mechanisms and supported neurotrophic factor production in the brain [82]. Such gut-to-brain immunochemical signaling can alleviate neuroinflammation and oxidative stress, which are predisposing factors to mood disorders and neurodegeneration [83]. The various action mechanisms are typically synergistic: the overall effect of simultaneous strengthening of epithelial barriers, the re-establishment of immune responses, and the strengthening of antioxidant status is the creation of the so-called anti-inflammatory and anti-oxidative environment where healing occurs [84].

## 4. Effects of Probiotics on Gut Inflammation and Gut Health

One of the main areas that probiotics have been beneficial is gut health. The in vitro gut models and human studies demonstrate that probiotics have the ability to suppress gastrointestinal inflammation and enhance the barrier function. *Lactobacillus* probiotics have the greatest potential as adjunctive therapies in IBD, which is a chronic relapsing inflammatory process of the gut [85]. In fact, *Lactobacillus* is a representative probiotic used for IBD management and is approved for alleviating ulcerative colitis. Therefore, studies confirm that administering *Lactobacillus* spp. can reduce colitic damage in mice (e.g., DSS-induced colitis) and improve symptoms in IBD patients [86,87]. Also, as revealed above, the probiotic treatment in colitis can enhance the intestinal immunological barrier and epithelial barrier function [8,88]. As an example, *L. casei* dieting in mice with colitis induced less inflammatory T cell infiltration in the colon because lactocepin mediated chemokine destruction causing a less severe inflammation [14,89]. Combinations of *Lactobacillus* with other probiotics or prebiotics have shown even greater efficacy in IBD. Another famous multi-strain mix (VSL3, which includes *Lactobacilli, Bifidobacteria, and Streptococcus*) has been shown to induce remission in patients with ulcerative colitis and in pouchitis clinical trials [90]. The above probiotic mix Bifico works remarkably well; in patients with IBD, the Bifico mixture reduced the disease activity, and the tight junction integrity of biopsy samples was restored [18]. These findings suggest that probiotic-induced improvements in gut immunology, through fortification of the intestinal barrier and modulation of mucosal immunity, have tangible clinical benefits for gut inflammation. Transient gut inflammation and dysbiosis not associated with IBD may also be alleviated through the use of probiotics. *Saccharomyces boulardii* and *L. rhamnosus* GG have been shown to reduce the duration of illness and decrease inflammatory cytokines in the gut in infectious or antibiotic-associated diarrhea, in part by replacing pathogens and preventing toxin-mediated NF-B activation [91]. With inflammation of a low grade and disturbed microbiota, such as in IBS, some probiotics have been indicated to have immunomodulatory effects [92]. One remarkable placebo-controlled trial of patients with IBS showed that, after 8 weeks, treatment with *Bifidobacterium longum* 35624 resulted in a significant reduction in symptoms (abdominal pain, bloating, etc.). Interestingly, the treatment also shifted the balance of circulating cytokines toward an anti-inflammatory profile [93]. In particular, an abnormal ratio at baseline of peripheral IL-10/IL-12 (imbalance between anti- and pro-inflammatory responses) was normalized in IBS patients with the help of *B. longum* 35624 through the enhancement of IL-10 secretion and/or a reduction in IL-12 secretion [94]. This is an indication that probiotics can also suppress inflammatory signals in non-severe gut pathology. A second study in IBS and ulcerative colitis patients observed that a probiotic yogurt (with *Bifidobacterium* and *Lactobacillus*) resulted in substantial reductions in systemic pro-inflammatory cytokines such as TNF-α in serum, indicating systemic effects of anti-inflammatory gut flora [95].

Notably, the gut probiotic application has symptoms on extra-intestinal organs through less systemic inflammation. Severe IBD is associated with elevated inflammatory mediators that can affect the liver and other organs; by calming gut inflammation, probiotics help prevent these downstream issues. The example of this is the lowering of gut inflammation by *Lactobacillus* in colitis, which has been associated with decreased incidence of IBD-related liver disease (such as autoimmune hepatitis) and even IBD-related arthritis and dermatologic flare [10,96,97,98]. Probiotics also alleviate oxidative stress in the gut, which can lower the mutagenic and cell-destroying environment. In a colon cancer prevention context, colitis-related colorectal cancer (CAC) can be induced by chronic colitis through the process of inflammatory oxidative DNA damage; positively, *Lactobacillus* interventions have been found to reduce the count and mass of tumors in mouse CAC models, in line with a reduction in the levels of IL-6, TNF-α, IL-1, and other tumor-promoting cytokines in the colon [99]. A particular anti-inflammatory agent of *L. casei* Shirota (polysaccharide–peptidoglycan complex) is said to suppress the IL-6/STAT3 signaling pathway in a CAC model, which suppressed tumor growth [100]. Although confirmation of cancer prevention in human trials is required, these findings corroborate the idea that anti-inflammatory effects of probiotics in the gut mucosa can be used to protect against the range of gut pathologies, including inflammatory diseases and neoplasia.

To conclude, probiotics (particularly *Lactobacillus* and *Bifidobacterium*) help to maintain gut health through restoring the gut barrier, correcting dysbiosis, and silencing inflammation. They inhibit the secretion of pro-inflammatory cytokines in the gut wall, enhance protective factors (mucus, SCFAs, antioxidant enzymes), and, therefore, disrupt the inflammatory cycle. In clinical terms, this is translated into better disease indices in IBD, quicker recovery from gastroenteritis, and even functional gut disorders. Oral probiotics interact first with the gut, and the capacity to generate an anti-inflammatory intestinal environment forms the basis of much of their systemic activity.

## 5. Effects on Neuroinflammation and the Gut–Brain Axis

The influence of probiotics on the gut–brain axis and neuroinflammation is one of the most promising areas in the field of probiotics. It is now known that gut microbes may affect brain physiology and behavior via immunological, neural, and metabolic mechanisms. Neuroinflammation can be encouraged by chronic inflammation of the periphery and increased gut permeability through the stimulation of microglia in the brain, and the reverse may occur with a reduction in central inflammation by probiotics [101,102].

Preclinical evidence provides proof-of-concept that specific probiotic strains can modulate neuroinflammatory and oxidative pathways. In a model of infection-induced cognitive impairment and depression-like behavior, *L. gasseri* NK109 administration was associated with decreased hippocampal IL-1β and increased brain-derived neurotrophic factor (BDNF), suggesting reduced neuroinflammatory signaling alongside enhanced neurotrophic support [19,103]. This result was in line with the suppression of depressive-like behaviors in mice, which indicated *L. gasseri* alleviated neuroinflammation and its behavioral outcome [103].

Another example is *L. rhamnosus* JB-1, which has been termed a psychobiotic because it possesses anxiolytic and antidepressant properties in rodents. It was demonstrated that *L. rhamnosus* JB-1 can regulate GABAergic signaling in the brain—amplifying the expression of GABA receptors in cortical areas and inhibiting them in limbic areas—eventually lowering anxiety and depression-like behaviors [104]. It is important to note that these effects on neurochemical processes require the presence of an intact vagus nerve, which emphasizes the importance of vagal gut–brain communication. Interestingly, in case scientists abluted CD4+CD25+, regulatory T cells, the positive outcome of *L. rhamnosus* JB-1 on behavior was never found, which implies that an immunologic factor (probably IL-10 produced by T regs) was needed to accomplish its psychotropic activity [79,105]. Therefore, *Lactobacillus* is able to induce immunoregulatory loops that eventually affect the brain.

Probiotics may also influence neuroactive metabolites and oxidative stress pathways. For example, certain L. plantarum strains can modulate tryptophan metabolism (potentially influencing serotonergic signaling), *L. brevis* can also synthesize GABA itself, and, as noted, *L. reuteri* can secrete the antioxidant amino acid, ergothioneine, which can cross the blood–brain barrier and has neuroprotective, anti-inflammatory effects [72,81,106,107]. The overall effect is typically a decrease in neuroinflammation and an enhancement in neural activity.

It has also emerged that probiotics may modulate pathways relevant to neuroinflammation in experimental models of neurodegenerative diseases; however, neurodegenerative disorders such as Alzheimer’s disease and Parkinson’s disease (PD) remain incurable, and the current evidence does not support probiotics as disease-modifying therapies.

In the neuroinflammatory processes that lead to the destruction of dopaminergic neurons in PD, increased intestinal permeability and gut dysbiosis have been implicated [108].

Some small clinical studies have reported improvements in gastrointestinal symptoms and reductions in selected inflammatory and oxidative stress markers during probiotic supplementation, occasionally accompanied by modest improvements in motor or non-motor outcomes [109,110]. As an illustration, patients who were given a multi-strain probiotic over 12 weeks had reduced serum TNF-α and MDA (a lipid peroxidation product) in addition to improvement in movement scores, compared with placebo [110]. These findings are logical considering the animal research studies in which probiotics inhibited microglial overactivation and dopaminergic neuron preservation; however, translation to clinical benefit remains uncertain [111]. While these observations are biologically plausible and consistent with preclinical data, available human studies are limited in size and duration, and probiotics cannot be considered therapeutic agents for PD. Similarly, in experimental models of AD, probiotic supplementation has been shown to decrease neuroinflammatory mediators, including IL-1β and TNF-α, and to modulate oxidative stress markers. Importantly, these findings are derived exclusively from in vivo and in vitro studies, and robust clinical trial evidence in patients with AD is currently lacking. Therefore, any discussion of probiotics in AD should be framed as mechanistic and hypothesis-generating, not as evidence of clinical benefit.

Some *Lactobacillus* and *Bifidobacterium* strains decreased the level of brain IL-1β and TNF-α and enhanced brain performance in AD mice, which was thought to occur through the restoration of gut microbial metabolites with anti-inflammatory actions in the brain (such as SCFAs); however, translation of these findings to clinical practice remains unproven [112]. Patients with AD were improving when being provided with probiotic milk during a 12-week period in terms of oxidative stress indicators and cognitive assessments, which suggests an effect on the neurodegenerative process in patients with AD (but data remain scarce) [113]. Interestingly, it has been proposed that *Lactobacillus* intervention in IBD has an indirect beneficial effect on AD and other CNS diseases that are facilitated by systemic inflammation [114]. Probiotics may alleviate inflammation that promotes neurodegeneration—one of the gut–brain axis crosstalk’s in chronic disease—by lowering the systemic inflammatory load (i.e., high circulating levels of IL-6, IL-1 linked to IBD) [115].

Another example that gives promise to probiotics is multiple sclerosis (MS), an autoimmune demyelinating CNS disease. MS is characterized by the destruction of the central nervous system by peripheral immune cells and abundant expression of inflammatory Th1/Th17 cytokines (IFN-7, IL-17, TNF) that contribute to neuroinflammation and neuronal injury [116]. The systematic review of MS clinical trials concluded that probiotic supplements may improve certain immune and inflammatory parameters of patients with MS [117]. The reviewed trials demonstrated the effect of multi-strain probiotics (usually *Lactobacillus* and *Bifidobacterium*) on patients with MS as a reduction in pro-inflammatory cytokines (such as IL-6, TNF-α) and an increase in anti-inflammatory cytokines (IL-10, TGF-beta) after 12–24 weeks of use [118]. Cases of reduction in oxidative stress indicators and an increase in the antioxidant status were also found in some studies [119]. Reported clinical outcomes (e.g., disability scores) have shown mixed or modest improvements and require confirmation in larger, well-controlled studies [120]. Though MS is a CNS disease, these effects are probably caused by probiotics influencing gut immunity (e.g., by generating regulatory T cells and anti-inflammatory gut macrophages), resulting in a more tolerogenic immune environment, which in turn influences the CNS. These results can be justified by the idea that the gut–immune brain axis is a valid therapeutic intervention in neuroinflammatory diseases: microbiota of the gut can be indirectly used to reduce the pathogenic response of the immune system in relation to the brain through its manipulation [118,121].

Overall, probiotics have shown an excellent ability to modulate the brain and behavior through the reduction in neuroinflammation and oxidative stress through the gut–brain axis. Among the proposed mechanisms are reductions in peripheral and CNS cytokines (IL-1b, TNF-α, IL-6, etc.), restoration of levels of HPA-axis and neurotransmitters, and even direct neuroprotective metabolite production [122]. While some human studies suggest that probiotics may have small-to-moderate effects on selected mood disorders (depression, anxiety) in specific populations, results are variable and strain-dependent, and the evidence base remains heterogeneous [123]. Even though human research has experienced its initial stage, initial experiments are optimistic. Probiotics as psychobiotics would prove to be an important addition to conventional therapies, in an attempt to soothe the immune system and oxidative environment that adds to the development of neurological disorders [124]. Of interest, future studies will determine the best strains and regimens to reduce neuroinflammation; yet, the current evidence strongly bears an indication of the gut microbial part in the control of brain inflammation and well-being. Crucially, probiotics should be regarded strictly as adjunctive nutritional supplements and must not be promoted as alternatives to evidence-based psychiatric or neurological treatments. Discontinuation of prescribed medications can be dangerous, and any probiotic use in these contexts should occur alongside standard medical care.

## 6. Systemic and Organ-Specific Impacts of Probiotics

The anti-inflammatory and antioxidant properties of probiotics are not limited to the gut and the brain, and this points to the possibility of probiotics being systemic therapeutic agents.

**Figure 2:**
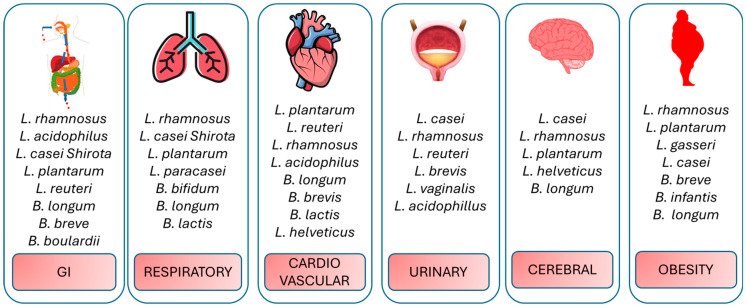
Role of probiotics in disease prevention and treatment.

Probiotics may help to increase the metabolic, hepatic, and cardiovascular health parameters, as well as other health parameters by lowering the levels of chronic low-grade inflammation and oxidative stress (Figure 2).

### 6.1. Metabolic Syndrome and Diabetes

Oxidative stress and chronic systemic inflammation are markers of type 2 diabetes mellitus (T2DM) and metabolic syndrome (MetS) [125]. Probiotic supplementation has been found to be of great help in this regard. Meta-analysis of 33 randomized trials of patients with diabetes indicated that probiotics had significant effects on decreasing circulating biomarkers of inflammatory responses and increasing antioxidant status [21]. It is worth highlighting that there were reductions in C-reactive protein (CRP, systemic inflammation), TNF-06 (systemic inflammation), and MDA (oxidative lipid damage) under the condition of probiotic treatment [21]. Simultaneously, antioxidant indices showing the most important antioxidants—glutathione (GSH) and total antioxidant capacity (TAC)—both increased in probiotic groups [21]. Such results indicate that there is a real decrease in the inflammatory/oxidative load in the bodies of patients. In keeping with this, another meta-review established that probiotics are likely to decrease IL-6 and hike anti-inflammatory IL-10 in individuals with metabolic conditions, but outcomes may depend on the strain and dosage [126]. Such changes in the clinical setting are not without meaning: decreased CRP and cytokines are associated with improved insulin sensitivity and a reduction in diabetes complications [127]. In fact, there are studies that have been made on patients with obesity or diabetes that have noted an improvement in insulin resistance and glucose levels, blood pressure, and liver enzymes following the use of probiotics, which has been attributed to the reduction in inflammation. As an example, a symbiotic yogurt of *L. plantarum* and *L. pentosus* strains was administered to adults with metabolic syndrome during 12 weeks in one placebo-controlled trial [128]. There was a significant increase in antioxidant enzyme activities (SOD and GPx) and TAC in the blood of the probiotic group, while total oxidant status was reduced compared with controls [128]. This increase in biochemicals was accompanied by small decreases in fasting glucose and triglycerides of the probiotic group, which is an expression of better metabolic health. Adipose tissue inflammation, which is one of the causes of insulin resistance, is also positively influenced: probiotics may suppress the expression of inflammatory adipokines in fat cells as well as reduce the infiltration of adipose tissue by macrophages [129]. The probiotics are identified through these effects as an additional treatment to decrease the chronic inflammation in MetS/T2DM and consequently minimize the risk of cardiovascular events [130,131].

### 6.2. Liver (Gut–Liver Axis)

Another organ that will benefit from the influence of inflammation caused by probiotics is the liver. The gut–liver axis concept is the basis behind non-alcoholic fatty liver disease (NAFLD) and liver fibrosis, in which subsequent hepatic inflammation is stimulated by augmented gut permeability and LPS influx [132]. Probiotics minimize the provision of pro-inflammatory stimuli to the liver by closing the gut barrier and regulating gut microbiota [133]. Research conducted in patients with NAFLD has revealed that probiotics are able to reduce serum liver enzymes—ALT (alanine aminotransferase) and AST (aspartate aminotransferase)—and markers of inflammation, and hence, liver injury. In a single study, patients with NAFLD taking a multi-strain probiotic showed a reduction in TNF-α and oxidative stress levels as well as increased insulin sensitivity compared with the control [134]. Probiotics also raise SCFAs such as butyrate that have anti-inflammatory consequences of the liver (butyrate is carried via the portal vein and can directly counteract the activation of hepatic stellate cells and inflammatory signaling) [135]. Probiotics have also been suggested to exert an adjunctive effect in more severe liver disease, e.g., in patients with liver cirrhosis, and probiotics have been shown to reduce circulating endotoxin and IL-6 levels, as well as the frequency of hepatic encephalopathy exacerbations, by modulating the gut microbiota away from ammonia-producing species [136]. *Lactobacillus* therapy in mice has been shown to lower liver inflammation scores in autoimmune hepatitis (an IBD-associated liver inflammation), presumably by stimulating regulatory immunity in the gut, which was transferred to the liver [137]. The overall picture of these findings is the systemic spillover of gut-based probiotic benefits, including the enhancement in gut integrity and immunity, resulting in reduced fuel to liver inflammation [138]. The outcome is a decrease in the hepatic environment, inducing less oxidative destruction of liver cells.

### 6.3. Cardiovascular System

Atherosclerosis and heart disease are known to be caused by chronic inflammation. The effect of probiotics in decreasing inflammatory mediators of the entire system could therefore be useful in cardiovascular health. In fact, it has been reported that serum CRP, IL-6, and lipid peroxides are reduced in several trials of probiotics in cardiovascular disease risk populations [139,140]. Indicatively, *L. fermentum* ME-3 is a strain with an impressive antioxidative activity, which, in a human study, has been found to decrease the oxidation level of LDL by approximately 20–30%, as well as increase the blood glutathione level of patients by approximately 16 percent [141,142]. These alterations allude to the fact that probiotics have the potential to enhance the oxidative profile associated with endothelial dysfunction. There is a relatively minor positive effect of some probiotics on the lipid profile (some *Lactobacillus* can absorb cholesterol or deconjugate bile acid, resulting in reduced LDL cholesterol) [143]. Probiotics have been shown to have small blood pressure and greater arterial compliance effects on hypertensive patients through the mitigation of inflammatory and dyslipidemic processes [144]. The other mechanism applicable to cardiovascular health is that gut microbes can decrease trimethylamine N-oxide (TMAO, a pro-atherogenic molecule) when exposed to it; an impending body of research is examining whether probiotics can positively modulate metabolism toward less TMAO [145,146]. Although probiotics do not substitute more mainstream cardiovascular therapies, they can be useful as an addition to reduce the overall cardiovascular risk since they have a multi-factorial effect on weight, glycemic control, blood lipids, and inflammatory markers [1,147,148].

### 6.4. Respiratory and Allergic Inflammation

Probiotics also have an immunomodulatory effect on the lung and allergic diseases (gut–lung and gut–skin axes). An example of how probiotics may influence the developing immune system towards hyper-inflammatory, Th2-skewed responses in children is that supplementation with *Lactobacillus* and *Bifidobacterium* strains during pregnancy and early life has been associated with a reduced prevalence of eczema and allergic rhinitis in children [4]. Several studies have reported a lower incidence of exacerbations and better pulmonary functioning when patients with asthma or chronic obstructive pulmonary disease (COPD) receive probiotics, possibly because probiotics would decrease systemic inflammation (reducing IL-17, IL-8, etc) and improve anti-viral defenses [149,150]. The gut–lung axis could be underpinned by the fact that probiotics may potentially boost anti-inflammatory cytokine IL-10 in the blood circulation and lung alveoli and reduce excessive immune response in the airways [151]. Furthermore, probiotics help in tightening the gut barrier, thereby cutting off the translocation of microbial products, which may be transported into the lungs leading to the development of inflammation [152,153]. Despite the inconclusive findings in respiratory diseases, there has been an overall tendency that the symptoms and biomarkers of inflammation are reduced in those who take probiotics during respiratory infections or allergic exacerbation of the disease than in non-users [154].

### 6.5. Other Systems

Probiotics have also been explored in renal, joint, and dermatologic diseases. Oral probiotics (alternatively known as gut microbiome modulators) in chronic kidney disease have the potential to decrease gut-derived uremic toxins and systemic oxidative stress, which can then retard the loss of kidney function [155,156,157,158]. Other pilot studies indicated that probiotics reduced serum endotoxin and inflammatory cytokines among dialysis patients [159]. In rheumatologic conditions such as rheumatoid arthritis, small studies of *L. casei* or multi-strain probiotics suggested a reduction in the number of tender joints and CRP, which showed an attenuation in joint inflammation [160]. In the same way, probiotics have been tested in systemic *Lupus erythematosus,* where some of them have been found to reduce the disease activity indices through immune modulation [161]. Another emerging field is dermatology: some *Lactobacillus* strains can be used topically or orally to reduce the severity of atopic dermatitis, which is likely to reduce systemic IgE and Th2 cytokines and enhance the skin barrier lipids [162,163,164]. Even implying the anti-inflammatory benefits of probiotics have been proposed to aid in acne or psoriasis (but strong evidence is yet to be found) [165,166].

The overall idea is that numerous chronic inflammatory conditions, whether it is the skin, the joints, or otherwise, are associated with dysbiosis in the gut and immune dysregulation, and, therefore, fixing the gut microbiome with probiotics may have far-reaching therapeutic implications [167,168]. In fact, scientists are currently considering the use of the so-called probiotic-derived molecules as inflammatory drugs. For example, a peptide of *L. helveticus* was discovered to inhibit NF-kB in human cells, and a pure *Faecalibacterium* metabolite (from a gut commensal often increased by *Lactobacilli*) was found to have strong anti-inflammatory properties in a colitis trial [169,170]. These results confirm the idea that the manipulation of commensal microbes can have a systemic anti-inflammatory effect.

It should be mentioned that the specificity of the strain plays a vital role; not every probiotic produces the same effect. Indicatively, *L. casei* Shirota can be particularly capable of stimulating IL-10 and decreasing IL-6, whereas *L. reuteri* ATCC 4659 is the only strain or mixture capable of upregulating HSPs in the gut [15,171]. Dosage and duration are also important; increased doses and interventions are likely to cause more pronounced alterations in the biomarkers of inflammatory/oxidative responses (to a certain extent) [172]. However, in most organ systems, we can identify a common pattern probiotic assist in the attenuation of inflammation and oxidative damage, promoting better clinical results or quality of life [31] (Table 3).

Thus, according to the data presented in Table 3, probiotic strains of *Lactobacillus* spp. exhibit multiple health-promoting effects, including improved outcomes in *Helicobacter pylori* eradication therapy, reduced incidence and duration of upper respiratory tract infections, lowered the risk of atopic eczema in infants, decreased abdominal visceral fat and body mass, alleviated depressive symptoms, and reduced the recurrence of bacterial vaginosis. These findings underscore the broad therapeutic potential of *Lactobacillus* spp. probiotics across gastrointestinal, metabolic, and immunological domains.

## 7. Probiotic Supplementation Safety, Limitations, and Possible Adverse Effects

As much as probiotics are seen to be safe and are largely consumed as dietary supplements or even as functional foods, there are no restrictions or possible dangers associated with their use. To balance and achieve a clinically relevant assessment of the probiotic interventions, the safety profiles, strain-specific variability, host factors, and conditions under which the probiotics may not be effective or even harmful need to be considered.

Probiotic supplementation is generally well tolerated in healthy people. Clinical trial adverse effects tend to be mild and short-lasting, most often comprising gastrointestinal effects (bloating, flatulence, abdominal pain, nausea, or bowel movements) [183]. The effects are commonly seen in the early days of supplementation and usually disappear automatically as the gut microbiota adjusts [184]. In randomized controlled studies, probiotics and placebos have a similar adverse event rate, indicating a general positive safety profile [185].

It should, however, be mentioned that the fact the general population does not reveal serious adverse effects does not mean that the drug is overall safe in all clinical situations [186]. The outcomes in which no quantifiable effect is identified regardless of the supplementation are also quite common and indicate that the effect of probiotics is not consistent and effective across all individuals and conditions [187].

Increased caution should be applied when probiotics are used in vulnerable populations, such as immunocompromised patients, critically ill patients, premature infants, elderly patients with severe comorbidities, and patients with disrupted intestinal barriers [188,189,190]. Serious but uncommon adverse events like bacteremia, fungemia (especially *Saccharomyces boulardii*) and sepsis have been reported, mostly in those with impaired immune systems, with central venous catheters, or patients with severe underlying conditions [191]. Translocation of probiotics through a defective intestinal barrier or contamination during administration could represent a clinically important threat in such populations. Hence, probiotic supplementation in immunocompromised or critically ill patients must not be regarded outside of medical supervision and must be chosen very carefully, with regards to strain, dosage, and monitoring [192].

The major weakness of probiotic studies and clinical translations is the excessive strain specificity. The strains of a single species can have significantly different impacts on immune regulation, oxidative stress, gut barrier, and tolerability [193]. This means that the findings on a particular strain cannot be applied to others, even within the same genus (e.g., *Lactobacillus* or *Bifidobacterium*) [194].

In addition, differences in formulation (single-strain vs. multi-strain products), dose, duration of administration, and quality of a product lead to mixed results in the studies [195]. Probiotics can have a neutral effect in certain situations, e.g., when the initial microbiota composition is balanced or when the probiotic strain used cannot colonize or survive in the host gut [196].

It is also not fully known that probiotic supplementation can be generalized to different ethnic groups and clinical conditions [197]. Baseline microbiota composition, geography, lifestyle, and medication use (e.g., antibiotics, immunosuppressants) are host-related factors that have a very strong impact on probiotic responsiveness [198]. There has been an early indication that people can also be categorized as responders or non-respondents to particular probiotic strains, based on host–microbiome interactions [199,200].

The majority of probiotic trials have been performed in restricted geographic areas and in rather homogenous populations, which limits the generalization of the results to more extensive and ethnically diverse populations [201]. This means that additional research is required to explain the efficacy of specific population, strain optimization, and individualized probiotic interventions.

## 8. Conclusions and Perspectives

The anti-inflammatory and antioxidant effects of probiotics, especially those of *Lactobacillus* spp., have proved to be convincing in various experimental and clinical conditions. Probiotics can damp down overreacting inflammation and enhance antioxidant defenses in the gut and the body by dampening microbiome modulation and interaction with immune and epithelial cells of the host. Some of the more important mechanisms are as follows: suppressing pro-inflammatory cytokine pathways (e.g., NF-B, MAPK), enhancing anti-inflammatory mediators (like IL-10 and TGF-β), strengthening of the intestinal barrier (tight junction upregulation and mucus production), reducing endotoxin load, and upregulation of the host antioxidant systems (SOD, catalase, glutathione).

Such actions are quantifiable primarily in terms of biological and inflammatory markers. In gastrointestinal disorders, probiotic supplementation may improve symptoms and inflammatory indices. In the context of the central nervous system, probiotics may influence neuroinflammatory and oxidative pathways and have been associated with modest effects on mood-related outcomes in selected populations. Importantly, probiotics should be regarded strictly as adjunctive nutritional supplements and not as substitutes for established pharmacological or psychological treatments, particularly in psychiatric or neurodegenerative disorders. Imperatively, the effects of probiotics are dependent on strain and context. Strains are not equally beneficial, and one might not react in the same manner. However, the evidence points to the fact that a carefully chosen combination of probiotic strains or mixtures of probiotics can be an effective addition to inflammatory disease therapies. They provide a comparatively safe method of tweaking the immune system, e.g., pushing an overactive immune system back to equilibrium instead of generally suppressing immunity (as used with drugs such as steroids). Another effect of probiotics is on oxidative stress, associated with the development of chronic inflammation, establishing a more conducive redox environment to promote tissue healing. The other benefit is that they act on multiple organs; affecting the gut microbiota, we can communicate to locations far away, such as the liver, skin, and the brain, through the immune and metabolic signaling pathways.

To continue, bigger, better controlled studies in particular groups of patients (e.g., in neurodegenerative diseases, in chronic kidney disease) are necessary to conclusively determine the level of probiotic effects. Treatment duration and dose optimization, as well as the discovery of responder subgroups, are the currently active research areas. Also, mechanistic understanding is going to expand further, i.e., studies of probiotic-derived metabolites (tryptophan metabolites, SCFAs, bacteriocins, probiotic antioxidants like ergothioneine) can provide new therapeutic molecules based on probiotics. Symbiotics (probiotics + prebiotic fibers) are also of interest to give synergistic effects on anti-inflammatory effects, since initial studies already indicate an added effect on colitis and cancer models.

To sum up, it is possible to state that the existing scientific data confirm the idea that probiotics can strongly influence anti-inflammatory and antioxidant impacts through the regulation of the gut–microbiome–host axis. These effects enhance the health of the gut and may be extended to improve systemic and neuroinflammatory conditions. Although probiotics are not a panacea, they are a promising and growing area of biotherapeutics, cooperating with the natural microbiome of the body to achieve health. As research continues to make probiotic interventions more tightly targeted, there is a high probability that these friendly bacteria will be the more systemic components of gastrointestinal tract to brain prevention and therapy strategies of inflammatory diseases. The idea to approach inflammation and oxidative stress with the help of microbiota regulation is a strong one, and it advocates in favor of the time-old saying that a healthy gut is the key to well-being.

## Figures and Tables

**Table 1:** Selected studies demonstrating the anti-inflammatory and antioxidant effects of probiotics.

Probiotic Strain(s)	Study Type	Target Condition/Tissue	Observed Anti-Inflammatory/Antioxidant Effects
*Bifidobacterium infantis* 35624	Human RCT (IBS patients)	IBS (gut)	Improved IBS symptoms; normalized IL-10/IL-12 cytokine ratio (↑IL-10, ↓IL-12), indicating enhanced anti-inflammatory immune balance [14].
*L. rhamnosus* (protein HM0539)	Animal (mouse colitis) + in vitro	Colitis (colon tissue)	HM0539 protein bound TLR4 and inhibited TLR4/MyD88/NF-κB signaling; ↓ COX-2 and iNOS expression; decreased NO and PGE_2_ levels [15].
*L. casei* (lactocepin-producing strain)	Animal (mouse colitis)	Colitis (colon tissue)	Secreted protease lactocepin, degraded chemokine IP-10, lowering T cell recruitment and significantly ameliorating colonic inflammation [16].
*Limosilactobacillus reuteri* ATCC PTA 4659	Animal (rodent colitis)	Ulcerative colitis (colon)	↑ Epithelial heat shock proteins HSP25/HSP70 expression; strengthened tight junction integrity; ↓ oxidative epithelial injury and colitis severity [17].
*L. gasseri* (Mn-SOD producing strain)	Animal (IL-10^−/−^ colitis model)	Colitis (colon tissue)	Elevated host antioxidant enzymes (Mn-SOD, CAT, GPx); ↓ neutrophil and macrophage infiltration; attenuated inflammatory damage [18].
*L. gasseri* NK109	Animal (*E. coli* K1-induced model)	Infection-induced neuroinflammation (brain)	↓ Hippocampal IL-1β and ↑ BDNF expression; improved neuroinflammatory status and depressive-like behavior [19].
Bifico (multi-strain: *L. acidophilus*, *Bifidobacterium*, *Enterococcus*)	Animal (mouse colitis) and human (IBD patients)	Colitis, IBD (gut)	↓ Colonic inflammation; ↑ ZO-1 and occludin expression; improved mucosal healing in animals and patients receiving Bifico [20].
Various probiotics (multi-strain; meta-analysis of 33 RCTs)	Human (meta-analysis; type 2 diabetes)	Type 2 diabetes (systemic inflammation)	↓ CRP, TNF-α, and MDA; ↑ glutathione and total antioxidant capacity; improved systemic inflammatory–oxidative profile and metabolic outcomes [21].
*L. plantarum* ZS62	Animal (mouse colitis)	Colitis, IBD (gut)	↓ The serum levels of MDA, MPO, IL-1*β*, IL-6, IL-12, TNF-*α*, and IFN-*γ* and the relative mRNA and protein expression of IL-1*β*, IL-12, TNF-*α*, COX-2, iNOS, and NF-*κ*B p65 in mouse colon tissues. Could inhibit colonic atrophy in IBD mice, reduce the degree of colonic damage [22].

Note: ↓—decrease; ↑—increase.

**Table 2:** Sources of probiotic metabolites and their antioxidative mechanism.

Bacterial Strain	Metabolite Produced	Antioxidative Mechanism	References
*L. acidophilus*	H_2_O_2_	Oxidation of parasitic proteins, inhibition of cysteine proteases	[53]
	Secondary metabolites (organic acids, amino acids, phenolics, terpenoids)	Activation of Nrf2/ARE, inhibition of JAK2/STAT3/MAPK, ROS reduction, modulation of AT1R/PPAR-γ	[54,55]
	Secreted/soluble metabolites	Caspase-3/8/9 activation, Bax/Bcl-2 → apoptosis; DPPH radical scavenging	[56]
*L. delbrueckii*	Secreted/soluble metabolites	Caspase-3/8/9 activation, Bax/Bcl-2 → apoptosis	[56]
*L. reuteri*	SOD, GPx, HO-1	ROS detoxification, mitochondrial stabilization, anti-inflammatory, hepatoprotection	[57]
	Intracellular enzymes and metabolites	Radical scavenging, ROS neutralization, intestinal and systemic antioxidant effects	[58]
*L. rhamnosus*	Bioactive peptides, exopolysaccharides (EPS)	↑SOD, ↑CAT, radical scavenging, anti-apoptotic, and intestinal protection	[59,60]
	EPS	Radical scavenging, metal chelation, anti-apoptotic, intestinal protection	[60]
*L. gasseri*	HO-1, CAT	Upregulation of antioxidant genes, DPPH radical scavenging, protection of HaCaT cells	[61]
	Nrf2, Sod1-3, Txn1, Hmox1, Nqo1, Gclc	Nrf2-ARE activation, enhanced enzymatic antioxidants, ROS reduction, cellular protection	[62]
	Radical scavengers, ferrous ion chelation	ROS reduction, oxidative protection	[63]
*L. casei*	SeNPs + GPx	Redox selenium combined with enzymatic support, antioxidative effect	[64]
	Inulin, phenolics	↑Enzymatic antioxidants, radical scavenging, metal chelation, anti-inflammatory	[65]
	GSH, proteins, lipids	Radical scavenging, redox balance, macromolecule protection	[66]
*B. bifidum*	Mn-SOD, catalase	ROS reduction, ↑antioxidant enzymes, intestinal protection	[67]
	Secreted/soluble metabolites	Radical scavenging, microbiota modulation, direct and indirect antioxidant effect	[68]
*B. longum*	SOD, GSH-Px, HDL	DPPH scavenging, ↓MDA, hepatoprotection, anti-aging, lipid regulation	[69]
	Genes for ROS scavenging	Oxygen tolerance, radical scavenging	[70]
	DAF-16 activation, NF-κB suppression	↓ROS, epithelial and immune protection, antioxidant effect independent of bacterial viability	[71]
*B. animalis*	Secreted/soluble metabolites	DPPH scavenging, ↓ROS, anti-lipid peroxidation	[72]

Note: ↓—decrease; ↑—increase.

**Table 3:** Systemic and organ-specific impacts of probiotics.

System/ Organ	Observed Effect (Clinical Outcome)	Lactobacillus Strain(s) Involved	Clinical Trial Evidence (Population, Context, Year)	References
Gastrointestinal	Symptom relief in IBS (reduced abdominal pain and bloating)	*L. plantarum* 299v (DSM 9843)	Four-week RCT in patients with IBS (2012) showed significantly less abdominal pain and bloating with *L. plantarum* 299v vs. placebo.	[173]
Gastrointestinal	Shorter duration of acute infectious diarrhea in children	*L.s rhamnosus* GG (LGG)	Meta-analysis of 11 RCTs in children (n = 2444, 2013) found LGG shortened diarrhea by ~1 day compared with placebo (mean 1.05 day reduction).	[174]
Gastrointestinal	Prevention of antibiotic-associated diarrhea	*L. rhamnosus* GG	Meta-analysis of 12 RCTs (2015) showed prophylactic LGG halved the incidence of antibiotic-associated diarrhea (overall risk ~22% → 12%; significant in children).	[175]
Gastrointestinal	Improved *Helicobacter pylori* eradication therapy outcomes (higher cure rate, fewer side effects)	*Lactobacillus* spp. (e.g., *L. reuteri*, *L. casei*, *L. acidophilus* as adjunct)	Meta-analysis of 30 RCTs (2016)—adding *Lactobacillus* probiotics to standard triple therapy increased H. pylori eradication rates by ~12–14% and reduced therapy-related diarrhea, nausea, and pain.	[176]
Gastrointestinal	Reduction in infant colic symptoms (less crying/fussing)	*L. reuteri* DSM 17938	Meta-analysis of 6 RCTs in infants with colic (2015)—*L. reuteri* significantly decreased daily crying time by ~43–46 min at 2–3 weeks of treatment vs. placebo.	[177]
Immune	Fewer upper respiratory tract infections (common colds) and shorter illness duration	*L. casei* strain Shirota (LcS)	Twelve-week RCT in healthy adults (2017)—daily LcS-fermented milk halved URTI incidence (22% vs. 53% in placebo) and shortened symptom duration, likely via immune modulation.	[178]
Skin (Dermatologic)	Lower risk of atopic eczema in infants (allergy prevention)	*L. rhamnosus* GG (ATCC 53103)	RCT in high-risk infants (mothers given LGG before birth, infants postnatal)—cumulative atopic eczema at age 2 was ~23% with LGG vs. 46% with placebo (relative risk ~0.5).	[179]
Metabolic	Reduced abdominal visceral fat and body mass (anti-obesity effect)	*L. gasseri* SBT2055 (LG2055)	Twelve-week RCT in adults with high BMI (2013)—daily fermented milk with *L. gasseri* led to ~8% reduction in visceral fat area and significant decreases in BMI, waist, and hip circumference vs. control.	[180]
Neurological	Improved depressive symptoms	*L. acidophilus* + *L. casei* (combined with *B. bifidum* in multi-strain formula)	Eight-week RCT in patients with major depression (2016)—probiotic group had a greater drop in Beck depression inventory scores (−5.7 points) vs. placebo (−1.5 points, *p* < 0.01).	[181]
Urogenital	Reduced recurrence of bacterial vaginosis	*L. crispatus* CTV-05 (LACTIN-V)	Phase 2b RCT in women with recent BV (2020)—vaginal *L. crispatus* probiotic after standard therapy lowered 12-week BV recurrence (30% vs. 45% with placebo, *p* = 0.01).	[182]

## Data Availability

Data sharing is not applicable.

## Abbreviations

The following abbreviations are used in this manuscript:

AD	Alzheimer’s Disease
ALT	Alanine Aminotransferase
APOE	Apolipoprotein E
AST	Aspartate Aminotransferase
ATCC	American Type Culture Collection
BDNF	Brain Neurotrophic Factor
CAC	Colitis-Related Colorectal Cancer
CAT	Catalase
CD	Cluster of Differentiation
CNS	Central Nervous System
COPD	Chronic Obstructive Pulmonary Disease
COX	Cyclooxygenase
CRP	C-Reactive Protein
DNA	Deoxyribonucleic Acid
DPPH	2,2-diphenyl-1-picrylhydrazyl
EDSS	Expanded Disability Status Scale
GABA	Gamma-Aminobutyric Acid
G-CSF	Granulocyte Colony-Stimulating Factor
GSH	Glutathione
GPx	Glutathione Peroxidase
HSP	Heat Shock Proteins
IBD	Irritable Bowel Disease
IBS	Irritable Bowel Syndrome
IFN	Interferon
IL	Interleukin
iNOS	Inducible Nitric Oxide
JAK	Janus Kinase
JNK	c-Jun N-terminal Kinase
LDL	Low Density Lipoprotein
LPS	Lipopolysaccharide
MAPK	Mitogen-Activated Protein Kinase
MDA	Malonaldehyde
MetS	Metabolic Syndrome
MPO	Myeloperoxidase
MS	Multiple Sclerosis
NAFLD	Non-Alcoholic Fatty Liver Disease
NF	Nuclear Factor
NO	Nitric Oxide
Nrf2	Nuclear Erythroid 2-Related Factor
PD	Parkinson’s Disease
PGE	Prostaglandin E
PTA	Probiotic Type Strain
RCT	Random Controlled Trial
RNA	Ribonucleic Acid
ROS	Reactive Oxygen Species
SCFA	Short-Chain Fatty Acids
SOD	Superoxide Dismutase
STAT	Signal Transducer and Activator of Transcription
T2DM	Type 2 Diabetes Mellitus
TAC	Total Antioxidant Capacity
TGF	Transforming Growth Factor
TFF	Trefoil Factor
TLR	Toll-Like Receptor
TMAO	Trimethylamine N-oxide
TNF	Tumor Necrosis Factor
ZO	Zonula Occludens
